# Effects of care burden on the life of caregivers of the elderly: A mixed-method study model

**DOI:** 10.1097/MD.0000000000030736

**Published:** 2022-10-28

**Authors:** Handan Sezgin, Seda Cevheroglu, Nur Demet Gök

**Affiliations:** a Health Sciences Faculty, Nursing Department, Eastern Mediterranean University, Famagusta, Turkey.

**Keywords:** care, care burden, elderly, nursing

## Abstract

Determining the care burden of elderly caregivers in the early period allows for early intervention to protect them from the negative physical, social, and psychological effects of care. This mixed-method study aimed to determine the burden levels of caregivers of elderly individuals and evaluate their opinions on the difficulties they experienced while caregiving. This study was conducted with caregivers of 89 elderly people who were determined to need care by visiting the homes of elderly people aged 65 years and over living in Famagusta. While the KATZ index of independence in activities of daily living and the Zarit Burden Interview tool were used to collect quantitative data, face-to-face interviews were conducted with 28 selected participants to collect qualitative data. The mean age of the caregivers was 52 ± 12 years; they were mostly female spouses/children/relatives, and 29.2% had moderate-to-severe care burden perceptions. The perception of caregiver burden levels increased as the level of dependency of elderly individuals and the duration of caregiving increased. Considering that caregiving burden affects every aspect of caregivers’ lives, it should be assessed regularly. To reduce care burden, it is recommended to expand home care services and short-term care facilities, use health technologies for continuous distance education and counseling in elderly care, and financially support caregivers who cannot work due to the responsibilities of providing elderly care.

## 1. Introduction

The 2019 Revision of World Population Prospects of the United Nations reported that the population aged 65 years and over would nearly double between 2025 and 2050 and that the share of the population aged 65-years-old and over would increase from 9% in 2019 to 16% in 2050.^[1]^ The elderly population in the Turkish Republic of Northern Cyprus (TRNC) is gradually increasing in parallel with the rise of this population taking place around the world. The State Planning Organization in the TRNC reported that the share of the elderly population increased from 8.12% in 2012 to 13% in 2017, indicating that the country’s population is aging.

Care is a fundamental human requirement. Elderly individuals experience biological, psychological, and physiological deterioration, noticeable inability to maintain their functions, and difficulties in their daily life activities. Limitations in daily life activities negatively affect elderly individuals’ quality of life and require care.^[2]^ Individuals who take on the responsibility of providing care to those unable to fulfill their basic needs on their own are required to maintain this care.^[3]^ In Turkey, family members largely meet the care needs of the elderly.^[4]^ In the TRNC, the care needs of the elderly with middle-to-high levels of income are met at their homes by foreign caregivers, but there are frequent changes in these caregivers due to culture- and language-related problems.^[5]^

However, during prolonged and unsupported caregiving, caregivers can face several challenges, including intense emotional distress, fatigue, sleep disorders, and difficulty maintaining the energy needed to provide care.^[6]^ Inadequately organized home care services have been shown to lead to more care burden, psychological stress, and emotional problems for those caring for the elderly.^[7]^ The role of health professionals has a significant impact on caregivers’ health and well-being. Nurses are the most important professional group in terms of training caregivers, increasing their motivation, and supporting them in the caregiving process.^[8]^

Since early detection of the burden on caregivers can protect them from the negative physical, social, and psychological effects of caregiving, this study aimed to provide insight into the difficulties experienced during the caregiving process and the burdens experienced by caregivers of the elderly and serve as a guide for further studies in this field.

## 2. Methods

This study was conducted using a mixed method design. The study was conducted in the Famagusta region of the TRNC from February 2016 to December 2017. Including 89 caregivers, all of whom met the following inclusion criteria: voluntarily agree to participate in the study, be 18 years of age or over, speak Turkish, have no communication-related problems, be the primary caretaker of the elderly person under their care, and be the primary caretaker of the elderly person under their care for at least 6 months. Those who refused to participate and those who were not at home during home visits were excluded from the study.

In this study, the descriptive features form created in line with the literature, the Zarit Burden Interview (ZBI), and the KATZ index of independence in activities of daily living (KATZ-ADL index) were used. Data were collected via face-to-face interviews conducted at the homes of elderly individuals. Each interview took approximately 30 to 45 minutes to complete. In the first stage of the study, sociodemographic characteristics and data related to the KATZ-ADL index were obtained from the elderly and their caregivers. In the second stage, the ZBI was filled out by the caregivers in a separate room. A questionnaire was administered to 28 female caregivers who agreed to answer the open-ended questions.

### 2.1. Data analysis

Data were inputted into BM SPSS Statistics, Version 20.0 (IBM Corp., Armonk, NY) and analyzed using descriptive tests. The *t* test, one-way ANOVA, Mann–Whitney *U* test, and Kruskal–Wallis test were used to compare the sociodemographic data against the scale scores. Linear regression analysis was used to assess the effects of the variables on caregiving burden, and Pearson’s correlation analysis was applied to assess the relationship between the scores on the two scales. The threshold for statistical significance was set at *P* < .05.

Raw qualitative data were created by the researcher participating in the interviews via the transfer of the participants’ responses to a Microsoft Word document. Two other researchers have read the transcripts several times. Content analysis was performed by considering the frequency of repeated words used by the participants, any comments they added to their responses, the number of participants making the same comment or using the same word, and the originality of the subjects they addressed the comments they made.

### 2.2. Ethical dimension

Before the study, ethics committee approval was obtained from the University of Research and Publication Ethics Board (2016/28-05). Written informed consent was obtained from the caregivers who agreed to participate in the study.

## 3. Results

The sociodemographic characteristics of the caregivers and elderly individuals are shown in Table 1. It was found that 42.7% of caregivers had mild levels of perceived burden.

**Table 1:** Sociodemographic characteristics of the caregivers and elderly individuals.

Variables	Number	%
Elderly		
Age, mean: 81 ± 7.78		
65–74	20	22.5
75–84	36	40.4
85 and more	33	37.1
Chronic diseases, mean: 2.80 ± 2.31		
Yes	82	92.1
No	7	7.9
Chronic diseases*		
Hypertension	54	60.7
Diabetes	41	46.1
Heart failure	33	37.1
Caregiver		
Age, mean: 52 ± 12 yr		
24–44	20	22.5
45–64	56	62.9
65 or higher	13	14.6
Gender		
Female	85	95.5
Male	4	4.5
Marital status		
Single	24	27.0
Married	65	73.0
Place of residence		
At home with their spouse	50	56.2
At home with their elderly relative	29	32.6
With their children	10	11.2
Employment status		
Employed	44	42.7
Retired	18	22.5
Housewife	27	34.8
Chronic diseases		
Yes	40	44.9
No	49	55.1
Chronic diseases*		
Hypertension	27	30.3
Diabetes	20	22.5
Heart disease	12	13.5
Articular and rheumatic diseases	12	13.5
Caregiver burden		
0–20 no	25	28.1
21–40 mild	38	42.7
41–60 moderate	24	27.0
61–88 severe	2	2.2
Mean = 31.49 ± 15.49, total	89	100

* Multiple selections were made.

Table 2 shows that the caregiving participants who had a chronic renal failure (*P* = .023) and were providing care to a dependent elderly person (*P* = .001) had higher scores on the ZBI. The participants who did not receive a formal education (*P* = .029), had income equal to expenses (*P* = .043), had been providing care for more than 5 years (*P* = .001), were the spouse of the elderly (*P* = .004), had articular and rheumatic diseases (*P* = .005), and asked for support with elderly care from their relatives (*P* = .001) or an institution (*P* = .002) also had higher scores on the ZBI. The participants expressed that they could not spare time for themselves (33.7%), that elderly person asks for more care than they need (14.6%), that caregiving harms their health (31.5%), that they experience difficulties managing their responsibilities related to their family, friends, and work (27.0%), that their social life is negatively affected (28%), and that they have financial problems (22.5%) had higher levels of perceived caregiver burden (*P* = .001).

**Table 2:** Comparison of the sociodemographic characteristics, difficulties experienced, and the ZBI scores of the elderly individuals and their caregivers.

Sociodemographic characteristics	Number	%	Score on the ZBI $\overset{⌣}{x}$	Test statistic
Elderly				
Chronic diseases*				
Chronic renal failure	8	9.0	37.75 ± 17.36	*t* = 2.368, *P* = .023
Dependency level of the elderly, mean KATZ ADL index score: 12.37 ± 4.35				
Independent^a^ (13–18)	50	56.2	21.92 ± 9.57	*F* = 58.276, *P* = .001 a < b < c
Semi-dependent^b^ (7–12)	27	30.3	39.37 ± 11.23	
Dependent^c^ (0–6)	12	13.5	53.66 ± 10.43	
Education level				
Literate^a^	7	7.9	37.57 ± 16.11	*F* = 3.721, *P* = .029 c < a
Primary or middle school^b^	40	44.9	35.20 ± 15.14	
High school or higher^c^	42	47.2	26.95 ± 14.77	
Caregiver				
Perception of income				
Less income than expenses^a^	14	15.7	25.14 ± 14.76	*F* = 3.293, *P* = .043 a < b
Income equal to expenses^b^	54	60.7	34.55 ± 14.77	
More income than expenses^c^	21	23.6	27.85 ± 16.38	
Duration of caregiving, mean: 5.16 ± 3.39 yr				
<5 yr	60	67.4	23.53 ± 10.26	*t* = −10.361, *P* = .001
>5 yr	29	32.6	47.96 ± 10.76	
Relationship to the elderly				
Spouse^a^	15	16.9	40.80 ± 16.40	χ^2^ = 13.116, *P* = .004 d < a
Child^b^	34	38.2	33.44 ± 15.19	
Relative^c^	7	7.9	36.42 ± 20.89	
Paid in-home caregiver^d^	33	37.1	24.21 ± 10.86	
Chronic diseases*				
Articular and rheumatic diseases	12	13.5	42.91 ± 16.83	*t* = 2.855, *P* = .005
Asks for support from their relatives				
Yes	31	34.8	39.41 ± 15.18	*t* = −3.472, *P* = .001
No	58	65.2	27.25 ± 14.03	
Asks for support from an institution				
Yes	16	18.0	42.37 ± 17.57	*t* = 3.267, *P* = .002
No	73	82.0	29.10 ± 14.03	
Thinks that the elderly ask for more care than they need				
No	61	68.5	26.34 ± 12.52	*t* = −5.290, *P* = .001
Yes	28	31.5	42.25 ± 15.71	
Thinks that they cannot make time for themselves				
No	59	66.3	26.57 ± 13.10	*t* = −4.669, *P* = .001
Yes	30	33.7	41.16 ± 15.46	
Thinks that they experience difficulties managing their responsibilities				
No	65	73.0	26.56 ± 12.94	*t* = −5.530, *P* = 001
Yes	24	27.0	44.39 ± 14.21	
Thinks that caregiving harms their health				
No	76	85.4	27.71 ± 13.16	*Z* = −10.011, *P* = .001
Yes	13	14.6	53.50 ± 7.90	
Think that their social life is negatively affected				
No	64	72.0	24.79 ± 11.29	*t* = −9.028, *P* = .001
Yes	25	28.0	48.37 ± 11.08	
Thinks that they have financial problems				
No	69	77.5	27.61 ± 13.04	*t* = −4.311, *P* = .001
Yes	20	22.5	43.50 ± 16.65	
Total	89	100		

Differences between the groups were determined through paired comparisons by using the Mann–Whitney *U* test after the results of the Kruskal–Wallis test performed with three or more groups became significant.

ZBI = Zarit Burden Interview.

* Multiple selections were made; *t*: *t* test; Z: Mann–Whitney *U* test; F: One-way ANOVA; χ^2^: Kruskal–Wallis test; *P* < .05.

A strong negative linear correlation was found between the scores on the KATZ-ADL index and those on the ZBI (*r* = −0.831, *P* = .001), meaning that the caregivers’ scores on the ZBI decreased as the dependency levels of the elderly increased (Table 3). Table 3 shows that the dependency of the elderly person, the duration of caregiving, and the education level of the caregiver explained 72.9% (*P* = .001) of the variance in caregiver burden. The levels of perceived caregiver burden increased as dependency on the elderly person (*R*^2^ = 69%; *P* = .001) and the duration of caregiving (*R*^2^ = 48%; *P* = .001) increased, while the perceived caregiver burden decreased as the education levels of the caregivers increased (*R*^2^ = 0.73%; *P* = .010).

**Table 3:** The variance among the dependency level of the elderly, duration of caregiving, and education level and burden of the caregiver.

Factors	*r*	*R* ^2^	*F*	*P*	beta	*t*	*P*	Adjusted *r*^2^	*F* change
KATZ ADL index score duration of caregiving ELoC		0.739	80.074	.000		10.660	.000	72.9%	80.074
KATZ ADL index score	0.831	0.690	193.648	.000	−0.644	24.453	.001		
Duration of caregiving	0.693	0.480	80.424	.000	0.262	6.944	.001		
ELoC	−0.270	0.073	6.863	.010	−0.084	−2.620	.010		

ELoC = Education Level of the Caregiver, KATZ-ADL index = KATZ index of independence in activities of daily living, *R*^2^ = linear regression analysis.

Table 4 shows some of the caregivers’ answers to questions about their experiences in the caregiving process.

**Table 4:** The caregivers’ responses to the questions on their experiences during the caregiving process.

Theme/subtheme	Some participants’ responses to the questions
Difficulties/*Physical, Psychological, Social*	**Participant (P24):** Even turning my father over in bed and sitting him down is a big deal. What’s more, I have intense lower-back pain from time to time; I cannot walk. There is no one to help me out.
	**(P12):** It is not easy to satisfy the elderly and meet their needs. I sometimes feel suffocated. I have had migraines for 2 yr. When it attacks, it last 2 d.
	**(P4):** I am providing care to my uncle. I am 76 years old, and he is 85 years old. He is not bedridden. The kids do the shopping, but even doing the household chores is too much for me. He keeps the television on all day and turns the volume way up because he cannot hear, but it drives me crazy! I get angry, my blood pressure goes up. I have been providing care to him for 8 yr. Now I need someone to provide care for me.
	**(P16):** I am not young either. I am taking care of two houses. My mother can barely move, and it is not easy to lift her out of the bed and lay her back down in the bed again. Thank God my father can look after himself. He also helps my mother. My back and legs ache, I cannot eat regularly, and my stomach also aches. I cannot even go to the doctor.
Inability to keep up with life/*Inability to fulfill family roles, Inability to cope with problems, Fatigue-Despair*	**(P7):** It takes up the entire day to take her to and from the hospital. We are living in the same house. She is watching my every movement. When I get dressed, she asks where I am going. It is like I, my husband, and my kids do not need anything. She gets sick when I plan to go out or when someone is going to visit us. I cannot make time for myself at all. It is really difficult to care for an elderly person.
	**(P14):** It is not easy to care for an elderly person. He is like a child. He misses his daughters and sons but mistreats me. He is never satisfied. I always give him everything he needs or asks for, but he never realizes that I am tired.
	**(P18):** I am working. My mother has diabetes, blood pressure issues, and heart valve problems. Her kidneys are not working well. She frequently needs to go to the hospital. Therefore, I have to take a day off. This makes me feel embarrassed, but what can I do? She is my mother. Our relatives sometimes help me, but the burden is still on me. How can I solve this problem?
	**(P21):** I do not have a regular family life any longer. I run between shopping, cooking, and cleaning all day. I have two kids; they are going to school. I cannot spare time for them. My husband and I are arguing too often. I feel too tired. There is no time to rest.
Need for support/*Increased duration of caregiving, Insufficient support with caregiving, Financial problems*	**(P2):** It has been 6 yr since my father had a stroke. My husband does not say anything, but I want to go on a holiday and spend time with my friends. I come here every day. My little brother comes once a month. If I had a sister, perhaps we could take turns, or if there was an institution, we could leave him there for 1 or 2 wk to allow me a break.
	**(P3):** Everything is too expensive. We have to buy both my mother’s and my father’s drugs. My father has a low salary. When we buy the drugs without prescriptions, we pay too much. We bought a saccharometer for my mother, but its sticks are expensive, and we cannot afford it. I know it is necessary for her health, but what can I do?
	**(P7):** I have four siblings, and all of them are working. I am the only one who is not employed, so I took my mother into my home to look after her. But I get tired. I want to go away sometimes, but my siblings do not care. They think that I am the best suited to watch her since I am not employed. I just want to spend time with my family at least 1 or 2 mo out of the year.
	**(P11):** My mother has Alzheimer’s disease. I have been providing care to her for 11 yr. She has not been able to move by herself for 2 yr. Do you think it is easy? I am taking care of her willingly, but every day is the same, and every year is worse than the previous one. I sometimes get angry and upset about something minor. I was not like that in the past. I want to get away from it all, even for a short time, but there is no one to whom I can leave the task of caretaking for my mother.
	**(P27):** My mother has been bedridden for 2 yr. Diapers are too expensive. We cannot afford it. We are trying to look after her with the salary from my father. We have to pay every time we take her to the hospital and also for her drugs.
Invisibility/*Damaged sense of justice, Unappreciated efforts for caregiving, Feeling of Comfort-Discomfort*	**(P3):** I visit my mother before going to my own home. I want her to stay with us, but neither she nor my husband wants this to happen. My mother says I took care of you, but you do not want to take care of me. I can’t keep up with the tasks. I become bad-tempered when I cannot meet my responsibilities. No one understands me. I have a bad feeling all the time.
	**(P5):** My husband says: “Share the caregiving responsibilities with your sister.” But my sister has a child with disabilities. I feel sorry for her. No one notices that I am rushing around and that I am tired. They also do not want to hear it. This is not fair.
	**(P9):** Everyone does what they want to do. My brother and his wife do not care at all. If you are complaining about it, you are the bad guy. If you do not talk about it, you are resigned to brooding. Since my mother is not bedridden, they think that I do not do anything. No one sees what I am doing.
	**(P25):** It has been 6 or 7 yr since I went on a carefree holiday. I think about leaving my parents to my brother and letting him take care of them, but I fear that he will always be asking me questions about everything related to caring for them. When I go somewhere, my mind is always at home. My body rests, but my heart is always restless.

## 4. Discussion

Turkish culture has a negative view of placing elderly people in nursing homes. Therefore, most female family members undertake caregiving responsibilities. Females who take on the role of caregivers express respect, love, and gratitude to their elderly relatives who raised them in this way.^[9,10]^ Timur et al and Unver et al^[10,11]^ found in their study that caregivers of the elderly were mostly female spouses and daughters. Similarly, nearly all caregivers in this study were female (95.5%) spouses and daughters of the elderly.

Participants in our study had mild levels of perceived caregiver burden. The ZBI scores of caregivers increased with increasing dependency on elderly individuals (*P* = .001). Studies in the literature support this finding.^[1,10]^ Previous studies have shown that an increase in the age and duration of caregiving, as well as a decrease in education level, degree of closeness with the elderly, decrease in income, the emergence of chronic diseases, and the inability to receive professional support, increase the caregiver burden of caregivers.^[9,12]^ Similarly, this study found that the caregivers who had lower levels of education and income, articular and rheumatic diseases, and who had been providing care for more than 5 years had higher levels of perceived caregiver burden (*P* ˂ .05), whereas the caregivers who were paid caregivers had mild levels of perceived caregiver burden (*P* = .004). In face-to-face interviews with female informal caregivers, they stated that they experienced physical, psychological, and social difficulties due to the extended duration of caregiving and that they needed both financial and emotional support for the care of the elderly. The number of elderly care centers in the TRNC where the study was conducted is insufficient, and there are no elderly care organizations to educate or support caregivers. It is believed that caregivers who do not receive adequate support experience a greater caregiver burden.

The presence of chronic diseases in elderly individuals is a factor that increases caregiver burden.^[13]^ A study conducted in Italy found that caregivers who did not receive support from their family members or social environment during the caregiving process had higher levels of caregiver burden.^[14]^ Similarly, this study found that 92.1% of the elderly and 44.9% of caregivers had chronic diseases. In addition, the caregivers expressed that their social life was negatively affected, that they could not make time for themselves, that they experienced difficulties managing the responsibilities for their family, friends, and work, that they needed support from their relatives, or that they had higher levels of perceived caregiver burden (*P* ˂ .05). The informal caregivers reported in the interviews that they had difficulties fulfilling their family roles, and thus felt helpless and tired all the time. These findings were supported by those reported in the literature.^[15,16]^

Studies on the burden of caregivers of individuals with chronic renal failure have shown that caregivers have higher levels of burden.^[16,17]^ The most common symptoms associated with articular and rheumatic diseases include pain, limitation of movement, stiffness, fatigue, and depression, which can lead to decreased working performance and limitations in daily life activities.^[18]^ Therefore, caregivers are more likely to have difficulties helping semi-dependent or dependent elderly individuals maintain their daily life activities due to pain, fatigue, and limitations in their activities these individuals experience, which can negatively affect the caregiver burden. Similarly, this study found that caregivers with articular and rheumatic diseases had higher levels of perceived burden (*P* < .05).

The main limitation of this study is that it can only be generalized to caregivers of elderly individuals in the Famagusta region.

## 5. Conclusion

For the care process to be carried out correctly and efficiently, it is very important to have home care services that will support caregivers and monitor the needs of the elderly. Furthermore, it is critically important that health and social state policies related to elderly care be reorganized. In this regard, it is recommended to create elderly care hotlines, develop videos and visual training materials, plan activities aimed at increasing the motivation of caregivers, and establish short-term care centers.

## Acknowledgments

We would like to thank Arzu Abiç, Sinem Dağ, Meltem Altinişik, Serpil Çağlayan, Dilek Karaoğlan, Gizem Düzenli and Serdar Aydin for their contributions to the “Health Profile, Quality of Life and Caregiving of Individuals aged 65 and over in the Famagusta Region” project.

## Author contributions

**Conceptualization:** Handan Sezgin, Seda Cevheroğlu, Nur Demet Gök.

**Data curation:** Handan Sezgin, Seda Cevheroğlu, Nur Demet Gök.

**Formal analysis:** Handan Sezgin, Seda Cevheroğlu, Nur Demet Gök.

**Funding acquisition:** Handan Sezgin, Seda Cevheroğlu, Nur Demet Gök.

**Investigation:** Handan Sezgin, Seda Cevheroğlu, Nur Demet Gök.

**Methodology:** Handan Sezgin, Seda Cevheroğlu, Nur Demet Gök.

**Project administration:** Handan Sezgin, Seda Cevheroğlu, Nur Demet Gök.

**Resources:** Handan Sezgin, Seda Cevheroğlu, Nur Demet Gök.

**Software:** Handan Sezgin, Seda Cevheroğlu, Nur Demet Gök.

**Supervision:** Handan Sezgin, Seda Cevheroğlu, Nur Demet Gök.

**Validation:** Handan Sezgin, Seda Cevheroğlu, Nur Demet Gök.

**Visualization:** Handan Sezgin, Seda Cevheroğlu, Nur Demet Gök.

**Writing – original draft:** Handan Sezgin, Seda Cevheroğlu, Nur Demet Gök.

**Writing – review & editing:** Handan Sezgin, Seda Cevheroğlu, Nur Demet Gök.
